# Tenofovir and kidney transplantation: case report 

**DOI:** 10.5414/CNCS108929

**Published:** 2016-08-29

**Authors:** Yuri Battaglia, Elena Cojocaru, Silvia Forcellini, Luigi Russo, Domenico Russo

**Affiliations:** 1 Department of Specialized Medicine, Division of Nephrology and Dialysis, St. Anna Hospital, University Ferrara, and; 2 Department of Public Health, Federico II University, Naples, Italy

**Keywords:** kidney transplant, chronic hepatitis B, tenofovir

## Abstract

Background: Hepatitis B viral infection (HBV) has been regarded as a contraindication for kidney transplantation because of the high risk of viral activation induced by immunosuppressive therapy. Anti-retroviral drugs have changed the prognosis of patients with hepatitis B viral infection (HBV+) who are candidates for renal transplant; indeed, therapy with antiretroviral drugs may ensure lower rates of morbidity and mortality compared to traditional therapies. Entecavir is the first-line antiviral therapy recommended for the treatment of HBV+ kidney-transplanted patients. In case of resistance to entecavir, tenofovir may be an alternative drug, either alone or in combination with entecavir. However, the best strategy of treatment is still unknown. In this case-report, a HBV+ kidney-transplanted patient who presented resistance to entecavir was initially treated by associating tenofovir to entecavir and with tenofovir alone afterward. This strategy induced complete remission of viral replication. Case presentation: In a HBV+ kidney-transplanted patient under monotherapy with entecavir, HBV flare (HBV DNA > 170.000 × 10^3^ UI/mL, HBeAg+, HbeAb–) occurred 9 months after transplantation; at that time, blood chemistry highlighted: creatinine 1.46 mg/dL, blood urea 65 mg/dL, e-GFR 50 mL/min, proteinuria 300 mg/24 h, calciuria 2,12 mmol/24 h, phosphaturia 0.56 g/24 h, vitamin D 11.5 ng/mL, PTH 130 pg/mL, calcemia 2.3 mmol/L, and phosphoremia 2 mg/dL. Liver elastometry (FibroScan) showed moderate fibrosis. Tenofovir was associated to entecavir. Three months after the combination therapy, reduction in HBV DNA replication (351 × 10^3^ UI/mL) was obtained. Creatinine and e-GFR were 1.48 mg/dL and 52 mL/min, respectively. At this point, entecavir was discontinued. After 13 months of tenofovir monotherapy, complete remission of viral replication was achieved but renal function deteriorated and proteinuria increased. Conclusion: This case-report indicates that tenofovir is effective in reducing viral replication of hepatitis B virus in a kidney-transplanted patient who presented resistance to previous treatment with entecavir. However, it should be taken into account that tenofovir could affect renal function.

## Background 

The number of patients with chronic hepatitis B viral infection (HBV+) is ~ 400 million all over the world [1, 2]. The prevalence of hepatitis B virus (HBV) infection in dialysis and renal transplant patients is considerably variable depending on different geographical areas [3]. 

HBV vaccination programs, the reduction of transfusions through the availability of erythropoietins, and the use of standard and special precautions for dialysis patients have reduced the number of HBV+ patients since the 1990s. At present, the incidence of HBV+ patients on kidney transplant waiting lists and transplant patients cohorts is 2.5% and 2.7%, respectively [4]. 

In the 1980s, Harnett et al. [5] highlighted that HBV+ patients on dialysis survive 5 years longer than kidney-transplanted patients (85 vs. 61%). Thus, the presence of HBV infection has been regarded as a contraindication for kidney transplantation for a longtime. Furthermore, the only available medication for treating HBV infection was interferon, which may cause acute rejection in transplant recipients [6]. 

In the second half of the 1990s, the availability of new antiretroviral drugs substantially changed the prognosis for HBV+ patients. These drugs cause lower rates of morbidity and mortality compared to traditional ones. 

Antiretroviral drugs are nucleoside, analogs that inhibit reverse transcriptase. Lamivudine was the precursor of all HBV antiviral drugs; but its use has not been very frequent because of the high drug-resistance rate (up to 53% after a 5-year therapy) [7]. 

By 2005, new nucleoside analogs, entecavir and telbivudine, and later on adenofovir and tenofovir, were approved for HBV infection therapy. Characteristic of these drugs was the low risk of viral resistance. 

Entecavir is mostly used in HBV+ renal post-transplant recipients because of its higher efficacy and lower incidence of viral resistance compared to lamivudine. 

In cases of resistance to entecavir, some authors suggest the combination of tenofovir (nucleotide analog) and entecavir (nucleoside analog) and to avoid the use of tenofovir as monotherapy [2, 8]. 

Tenofovir was approved in 2001 by the (U.S.) Food and Drug Administration (FDA) as an antiretroviral drug for Human immunodeficiency virus (HIV) infection. In 2008, the FDA approved tenofovir in HBV+ patients. Recent studies [9, 10, 11, 12, 13, 14] have demonstrated the efficacy of tenofovir either alone in case of HBV resistance to lamivudine or in combination with entecavir in cases of multidrug resistance. 

Tenofovir is a reverse transcriptase inhibitor. Tenofovir is eliminated through the kidneys, 80% via glomerulus and 20% through the proximal tubule [15]. The more frequent side effects are gastrointestinal alterations, bone demineralization with osteopenia and/or osteoporosis, acute pancreatitis, Fanconi syndrome, tubular necrosis, and acute interstitial nephritis [16, 17]. Tenofovir interacts with two organic anion transporters (hOAT1 – hOAT3) placed on the basolateral membrane of the proximal tubule. Moreover, tenofovir interferes with MRP-2 and MPR-4 carriers inducing mitochondrial DNA damage [18, 19]. Nephrotoxic drugs, diabetic nephropathy, HIV – HBV coinfection, arterial hypertension, age over 50 years, and liver cirrhosis increase the risk of tenofovir nephrotoxicity [20]. 

While many data are available on patients with either HBV or HIV infection [22, 23], very few cases of kidney-transplanted patients who have been treated with tenofovir have been reported [10, 21]. In addition, there is only one case report reported in the literature of a kidney-transplanted recipient on therapy with tenofovir resistance to entecavir [21]. 

Herein, we describe the case of a HBV+ kidney-transplanted patient with acquired resistance to entecavir, who initiated combination therapy with entecavir and tenofovir and was successively maintained on mono-therapy with tenofovir. This strategy allowed for the achievement of complete remission of HB virus replication. 

## Case presentation 

In April 2010, a 58-year-old male was hospitalized for advanced chronic renal insufficiency (e-GFR 6 mL/min). His clinical history showed arterial hypertension, insulin-dependent type II diabetes mellitus, and HBV infection. The latter had been detected in 2000. 

One month later, the patient started hemodialysis. Blood chemistry highlighted: HBeAg +, HBsAg +, HBsAb - e HBV DNA 8.620 × 10^3^ UI/mL, AST 19 U/L, ALT 27 U/L, LDH 293 U/L, GGT 130 U/L, ALP 233 U/L, alpha-fetoprotein 3.7 ng/mL, bilirubin 0.29 mg/dL, total protein 7.6 g/dL, albumin 4.5 g/dL, white blood cells 5.58 × 10^3^/µL, and platelets 91 × 10^3^/µL. 

Abdominal ultrasonography showed mild enlargement of liver and spleen (longitudinal diameter 157 mm) with normal structure and no sign of focal lesions. These findings suggested the diagnosis of chronic liver disease secondary to HBV infection. The patient refused to undergo the suggested liver biopsy. 

**Figure 1.**
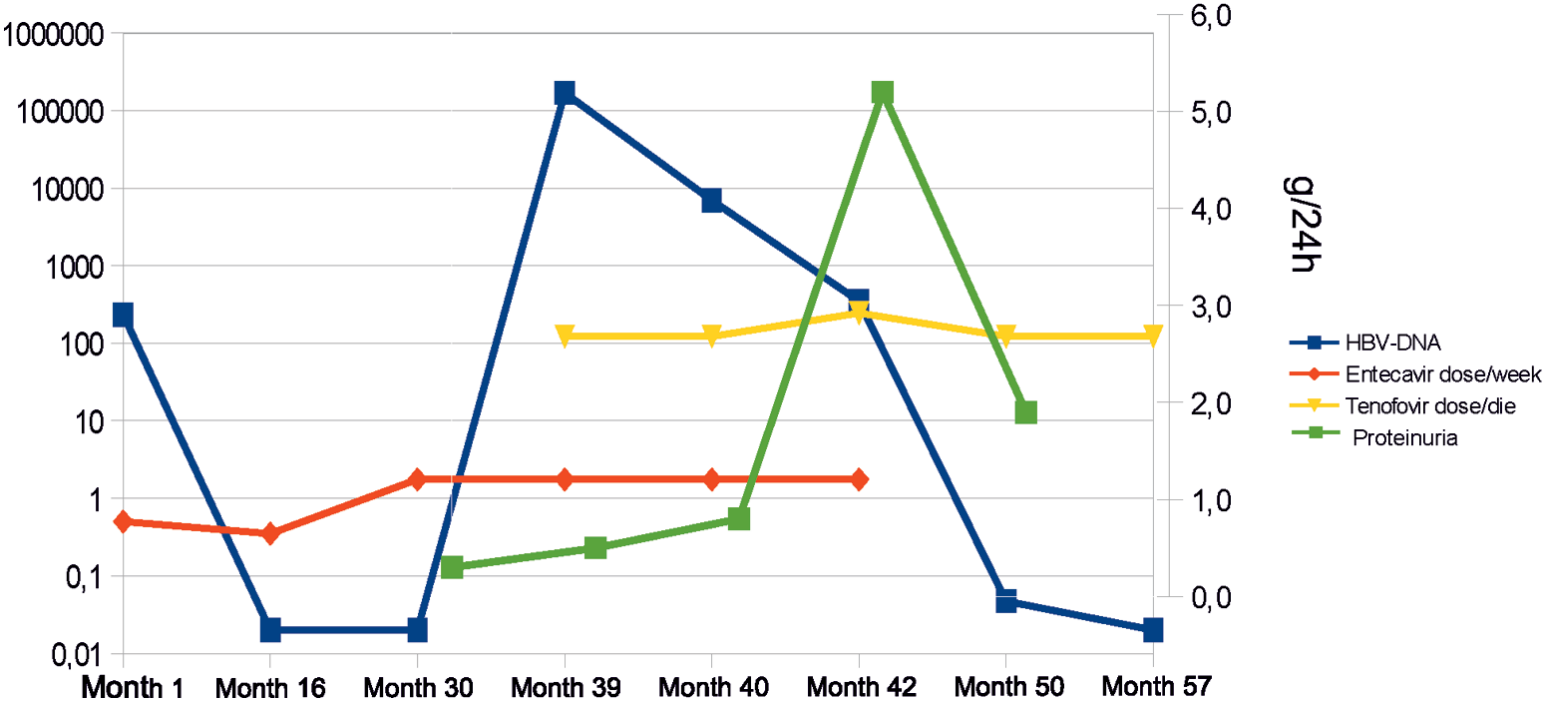
Hepatitis B virus DNA and proteinuria during entecavir and tenofovir therapy: Entecavir was started in month 1 for HBV flare. Hepatitis B virus DNA (HBV-DNA) slowly reduced and was absent in month 16. Viral load rebound presented in month 39 so tenofovir was added to entecavir at this time point. In month 42, entecavir was stopped, and viral load continued to decrease gradually.

In January 2011, 8 months after the beginning of hemodialysis, laboratory tests showed: Hepatitis B virus DNA (HBV-DNA) 47.900 × 10^3^ UI/mL, HBsAg +, HBsAb-, HBcAb +, HBeAg+, HBeAb–, ALP 248 U/L, LDH 340 U/L, GGT 133 U/L, total bilirubin 0.62 mg/dL, ALT 79 U/L, and AST 54 U/L. The patient started entecavir 0.5 mg per week as single dose at the end of hemodialysis session. This time point was regarded as month 1 (Figure 1). 

After a 3-month therapy with entecavir, chemistry showed: HBV-DNA 235 × 10^3^ UI/mL. HBsAg +, HBsAb-, HBcAb +, HBeAg+, HBeAb-, ALP 185 U/L, LDH 329 U/L, GGT 78 U/L, total bilirubin 0.4 mg/dL, ALT 27 U/L, and AST 20 U/L. The liver and spleen ultrasound evaluation was unchanged. 

One year later (month 15), the HBV-DNA was < 20 UI/mL with stability of all others markers (HBsAg +, HBsAb-, HBcAb +, HBeAg+, HbeAb-, ALT 9 U/L, and AST 11 U/L.). Thus, entecavir was given at a dose of 0.5 mg every 10 days. 

In month 29, the patient underwent successful cadaveric donor renal transplantation. Immunosuppressive therapy with tacrolimus and everolimus at throw levels of 4 – 6 ng/mL and 3 – 8 ng/mL, respectively, was started. One month after transplantation, blood chemistry showed: creatinine 1.39 mg/dL, blood urea nitrogen 57 mg/dL, e-GFR (CKD-EPI) 53 mL/min, proteinuria 500 mg/24 h, AST 11 U/L, ALT 71 U/L, total bilirubin 1.12 mg/dL, and HBV DNA < 20 UI/mL. The dose of entecavir was increased to 0.5 mg a day to avoid the activation of HBV. 

The patient did not observe the standard follow-up. Nine months after transplantation (month 38), biochemistry showed: HBV DNA > 170.000 × 10^3^ UI/mL, HBeAg+, HbeAb–, creatinine 1.46 mg/dL, blood urea 65 mg/dL, e-GFR 50 mL/min, proteinuria 300 mg/24 h, calciuria 2,12 mmol/24 h, phosphaturia 0,56 g/24 h, vitamin D 11,5 ng/mL, PTH 130 pg/mL, calcemia 2,3 mmol/L and phosphoremia 2 mg/dL. 

Considering the increase in viral replication, we decided to add tenofovir (245 mg/48 h) to entecavir. During the 1^st^ month (month 39) of double antiviral therapy, HBV replication decreased (HBV DNA 6.960 × 10^3^ UI/mL), with ALT (42 U/L) and AST (36 U/L). GFR, phosphorus, and phosphaturia remained stable. Liver elastometry (FibroScan) showed moderate fibrosis. 

After 3 months of association therapy, there was further reduction in HBV DNA (351 × 10^3^ UI/mL). Creatinine and e-GFR were 1.48 mg/dL and 52 mL/min, respectively. Entecavir was suspended, and tenofovir was increased to daily dose of 245 mg. 

After 8 months (month 49) of mono-therapy with tenofovir, HBV DNA further decreased (0.047 × 10^3^ UI/mL). However, decline of kidney function (creatinine: 2.05 md/dL and e-GFR: 35 mL/min) and increase of proteinuria (from 970 mg/24 h to 5,200 mg/24 h) was observed. Biochemistry showed: total proteins 6.6 g/dL, albumin 56%, alpha-2-globulin 17%, gamma globulin 12,2%, total cholesterol 94 mg/dL, calciuria 3 mmol/24 h, phosphaturia 0.88 g/24 h, PTH 95.7 pg/mL, albumin-corrected serum calcium 2.3 mmol/L, phosphorus 2.6 mg/dL, and vitamin D: 8.7 ng/mL. The dose of tenofovir was reduced to 245 mg/48 h according to e-GFR. Kidney biopsy was performed; histology showed: tubular atrophy, interstitial fibrosis, and arterial fibro-intimal thickening in absence of signs of graft rejection. There were no anti-HLA antibodies. Everolimus was stopped and therapy with mycophenolic acid (dose of 360 mg twice daily) was started. Five months after the initiation of this scheme (month 54), HBV viral DNA completely disappeared. Renal function remained unchanged (creatinine: 2.15 mg/dL; e-GFR: 32 mL/min), while proteinuria progressively declined with the lowest levels of 1.9 g/24 h recorded on month 57. 

## Discussion 

Nucleoside and nucleotide inhibitors (NOs) are safer and more effective than interferon alpha in treating HBV infection in kidney-transplanted patients despite the fact that they may cause kidney damage and may induce single or multiple resistances during prolonged treatment [9, 10]. 

Entecavir is the preferred therapy for kidney transplant patients. Entecavir may be used either as first-line therapy or as substitute therapy when resistance to lamivudine or to adefovir occurs. Entecavir reduces virus replication without negative effects on GFR and proteinuria and without increasing the risk of graft rejection [24, 25, 26, 27, 28]. 

In our case report, entecavir alone induced complete suppression of virus replication when the patient was on dialysis. In contrast, entecavir was unable to avoid HBV flare during the post-transplantation phase despite the incremented dosage. This finding underlines that immunosuppressive therapy after organ transplantation may influence the response to entecavir. 

In cases of HBV flare in kidney-transplanted patients, the best treatment strategy is still unknown. It is debated whether it is more appropriate to replace the antiviral drug in use with a different one or coadministering drugs with different mechanisms [21]. The former option carries the risk of multidrug resistance [2, 29]. The latter option seems more effective; indeed Shan et al. [21] reported a more powerful antiviral effect by combining tenofovir with entecavir compared to combination of adefovir with entecavir. In addition, the combination of tenofovir and entecavir did not cause renal damage. 

In our patient, entecavir was slowly tapered up to its discontinuation; tenofovir was associated to entecavir during a 3-month association period, and it remained as mono-therapy. 

Tenofovir has been used less frequently than entecavir in kidney transplant patients due to its possible nephrotoxic side effects. Some authors reported that 15% of patients treated with tenofovir may have proximal tubule dysfunction (Fanconi syndrome, isolated and persistent hypophosphatemia), acute renal insufficiency, or irreversible renal damage. Indeed, these alterations have been described even after 9 years from the initiation of therapy [30, 31, 32]. 

It is worth mentioning that in the present case, the decreased renal function and the increased proteinuria appeared a few months after monotherapy with tenofovir. Renal biopsy provided no unequivocal histological evidence; unfortunately, electronic microscopy was not performed. Lesions could have likely been caused either by everolimus (proteinuria and vascular damage) or by tenofovir. The latter may worsen tubular atrophy and interstitial fibrosis when risk factors such as diabetes and middle age are present [23, 33]; our patient was middle-aged and diabetic (since year 1990). Finally, additive negative effects of both everolimus and tenofovir on kidney function could not be excluded. 

Despite the concerns about likely tenofovir-dependent side effects on renal function, the drug was not withdrawn in order to not expose the patient to the risk of viral reactivation. Monotherapy with tenofovir ensured a long-lasting control of virus replication; relapse and further decline of renal function were not observed during the following 16 months. 

## Conclusions 

Hepatitis B viral infection may still represent a relevant problem for kidney-transplanted patients despite the improved prognosis obtainable with the new antiretroviral medications. 

Herein, the reported case indicates that tenofovir is effective as monotherapy in controlling HBV infection in the case of resistance to therapy with entecavir in a kidney-transplanted patient. Potential nephrotoxicity of tenofovir may not be excluded. It is mandatory to strictly monitor changes of renal function and proteinuria during therapy with tenofovir. 

## Acknowledgments 

None. 

## Informed consent 

Informed consent was obtained from the case report participant described in the study. 

## Ethical approval 

All the procedures performed in this study involving human participants were in accordance with the ethical standards of the institutional and/or national research committee and with the 1964 Helsinki declaration and its later amendments or comparable ethical standards. 

## Conflict of interest 

The authors declare that they have no conflict of interest.
